# Supplementary material for: The Impact of Race and Age on Response to Neoadjuvant Therapy and Long-Term Outcomes in Black and White Women with Early-Stage Breast Cancer
Source: Res Sq. 2023 Mar 16:rs.3.rs-2667554. Preprint. [Version 1] doi: 10.21203/rs.3.rs-2667554/v1 (PMC10055663; doi:10.21203/rs.3.rs-2667554/v1)
Supplement: Supplement 1 [file NIHPPrs2667554v1-supplement-1.pdf]

Suppl Table 1. Association Between Race and Age and Disease-Free and Overall Survival for Women Treated with Neoadjuvant Chemotherapy

	Disease-free Survival				Overall Survival			
	Univariable Analysis		Multivariable Analysis*		Univariable Analysis		Multivariable Analysis*	
	HR (95% CI)	P-value	HR (95% CI)	P-value	HR (95% CI)	P-value	HR (95% CI)	P-value
**young Black vs. young White**	2.19 (1.11-4.32)	0.023	1.80 (0.83-3.91)	0.139	2.22 (0.94-5.29)	0.070	1.56 (0.57-4.26)	0.386
**young Black vs. older Black**	0.99 (0.57-1.73)	0.981	0.80 (0.43-1.51)	0.498	0.78 (0.40-1.52)	0.463	0.66 (0.31-1.40)	0.280
**young White vs. older White**	0.86 (0.44-1.68)	0.663	0.73 (0.35-1.54)	0.414	0.92 (0.38-2.21)	0.845	0.92 (0.36-2.61)	0.957
**older Black vs. older White**	1.90 (1.10-3.28)	0.020	1.64 (0.92-2.93)	0.096	2.62 (1.31-5.26)	0.007	2.31 (1.10-4.82)	0.026

Abbreviations: 95% CI, 95% Confidence Interval
*adjusted for stage, grade, subtype, and Charlson Comorbidity Index

**Suppl Table 2. Neoadjuvant Chemotherapy Trial Enrollment by Race and Age**				
	Odds Ratio of Enrolling in a NACT Trial Compared to Young White Women			
	Unadjusted Model		Adjusted Model*	
	**OR (95% CI)**	**P-value**	**OR (95% CI)**	**P-value**
**young Black**	0.43 (0.15-1.21)	0.111	0.61 (0.17-2.16)	0.446
**older Black**	0.34 (0.14-0.80)	0.013	0.65 (0.23-1.85)	0.229
**older White**	0.87 (0.44-1.72)	0.682	1.24 (0.50-3.13)	0.653

*adjusted for stage, subtype, grade, and Charlson Comorbidity Index

**Suppl Table 3. Clinical Trial Enrollment by Race and Age**				
	Odds Ratio of Enrolling in a Trial Compared to Young White Women			
	Unadjusted Model		Adjusted Model*	
	**OR (95% CI)**	**p value**	**OR (95% CI)**	**p value**
**young Black**	0.40 (0.18-0.88)	0.024	0.34 (0.13-0.90)	0.030
**older Black**	0.31 (0.19-0.52)	0.000	0.53 (0.29-0.99)	0.045
**older White**	0.46 (0.29-0.73)	0.001	0.85 (0.48-1.53)	0.595

*adjusted for stage, subtype, grade, and Charlson Comorbidity Index
